# Fine specificity of anti-MSP1_19 _antibodies and multiplicity of *Plasmodium falciparum *Merozoite Surface Protein 1 types in individuals in Nigeria with sub-microscopic infection

**DOI:** 10.1186/1475-2875-9-287

**Published:** 2010-10-18

**Authors:** Josiane Ngoundou-Landji, Roseangela I Nwuba, Chiaka I Anumudu, Alexander B Odaibo, Wenceslas D Matondo Maya, Henrietta O Awobode, Christian M Okafor, Olajumoke A Morenikeji, Adanze Asinobi, Mark Nwagwu, Anthony A Holder, Francine Ntoumi

**Affiliations:** 1 Université des Sciences et Techniques de Masuku, Franceville, Gabon; 2 Cellular Parasitology Programme, Department of Zoology, University of Ibadan, Nigeria; 3 Unité de Recherches Médicales, Hôpital Albert Schweitzer, Lambaréné, Gabon; 4 Department of Parasitology, Institute for Tropical Medicine, University of Tübingen, Germany; 5 Fondation Congolaise pour la Recherche Médicale/Université Marien Ngouabi, Republic of Congo; 6 College of Medicine, University of Ibadan, Nigeria; 7 College of Arts and Science, Northwest University, Kirkland WA 98033, USA; 8 Division of Parasitology, MRC National Institute for Medical Research, The Ridgeway, Mill Hill, London NW7 1AA, UK

## Abstract

**Background:**

The absence of antibodies specific for the 19 kDa C-terminal domain of merozoite surface protein 1 (MSP1_19_) has been associated with high-density malaria parasitaemia in African populations. The hypothesis that a high prevalence and/or level of anti-MSP1_19 _antibodies that may inhibit erythrocyte invasion would be present in apparently healthy individuals who harbour a sub-microscopic malaria infection was tested in this study.

**Methods:**

Plasma samples were collected from residents in a region in Nigeria hyperendemic for malaria, who had no detectable parasitaemia by microscopy. Using a competition-based enzyme-linked-immunosorbent assay with two invasion-inhibitory monoclonal antibodies (mAbs) 12.10 and 12.8, the levels and prevalence of specific antibodies were measured. The minimum multiplicity of infection was determined using PCR. The prevalence of anaemia was also measured.

**Results:**

Plasma samples from 85% of individuals contained antibodies that bound to MSP1_19_. The inhibition of mAb 12.10 binding was strongly correlated with the prevalence (Spearman correlation test, p < 0.0001) and mean titre of anti-MSP1_19 _antibodies (Spearman correlation test, p < 0.001) in the samples. Comparing samples from individuals with multiple infection (group M) and single infection (Group S), group M contained a higher (p = 0.04) prevalence of anti-MSP1_19 _antibodies that competed with mAb 12.10. Using a logistic regression model, it was found that the presence of antibodies competitive with mAb 12.10 was affected negatively by anaemia (p = 0.0016) and positively by the carriage of multiple parasite genotypes (p = 0.04).

**Conclusions:**

In the search for correlates of protection against malaria, which will be essential to evaluate clinical trials of malaria vaccines based on MSP1, this study examines some potential assays and the factors that need to taken into account during their evaluation, using samples from individuals naturally exposed to malaria infection.

## Background

Among many *Plasmodium falciparum *merozoite surface antigens, merozoite surface protein (MSP) 1 has been shown to be one of the major targets of antibodies that inhibit the invasion of red blood cells [1-3]. The protein is present on the merozoite surface as a complex of polypeptides that includes a glycosylphosphatidyl inositol (GPI)-anchored 42 kDa C-terminal fragment (MSP1_42_). During merozoite invasion into the red blood cell MSP1_42 _is processed to yield 33- and 19-kDa fragments (MSP1_33 _and MSP1_19_, respectively). Only the GPI anchored MSP1_19 _remains on the merozoite during erythrocytes invasion [4,5]. Some mouse monoclonal antibodies (mAbs) including 12.8 and 12.10 [6] that inhibit the invasion of red blood cells also inhibit the processing of MSP1_42 _[7]. However, it is still not clear that this activity is a significant contribution to protective immunity acquired following exposure to the parasite [8].

Several immuno-epidemiological studies have yielded conflicting results with regards to the association between anti-MSP1_19 _antibodies and protection against clinical malaria [9-15]. At least one study [16] has indicated that the total anti-MSP1_19 _antibody titre is a poor indicator of malaria immunity, suggesting that antibody fine specificity is very important. It has been proposed that functional assays such as growth inhibition assays [17], inhibition of MSP1_42 _processing [2] or Fc-mediated effector mechanisms [18] may provide a more informative readout to identify useful antibodies. The fine specificity of such functional antibodies may be examined using a numbers of methods including direct binding to antigen or modified antigen [19,20] or competition assays using defined mAbs [21-23]. Using these approaches different classes of antibody have been defined and their epitopes partially mapped; for example MSP1_42 _processing and merozoite invasion inhibitory antibodies, blocking antibodies that block the activity of invasion inhibitory antibodies, and neutral antibodies that have no effect on MSP1_42 _processing and merozoite invasion [2,8,20,24].

MSP1_19_-specific invasion inhibitory activity has been associated with resistance to reinfection in Kenya [25]. However, parasite inhibitory activity is limited to a small subset of total anti-MSP1_19 _antibodies. mAbs 12.8 and 12.10 have been used in several sero-epidemiological studies [16,21,23]. In one study in The Gambia [21], individuals with anti-MSP1_19 _antibodies that compete with mAb 12.10 in a specific ELISA, were significantly less likely to have malaria infections with densities of ≥ 1,000 parasites/μl. In a study in Uganda, competition with mAb 12.10 was highly correlated with resistance to high-density parasitaemia, but there was no such association with mAb 12.8 [23]. The precise epitope mapping of both mAbs has been reported recently [26,27] and although there is considerable overlap of the two epitopes the data above suggest different functions for the corresponding antibodies [26].

A previous study carried out in the rural area of Igbo-Ora, South-western Nigeria [16] showed no correlation between the level of naturally acquired anti-MSP1_19 _antibodies and inhibition of MSP1_42 _processing in plasma samples from *P. falciparum *infected children and adults. To test the hypothesis that in apparently healthy individuals who harbour sub-microscopic malaria parasite infections there would be a high prevalence and/or high level of anti-MSP1_19 _antibodies that compete with mAbs 12.8 and 12.10, further investigations in the Igbo-Ora population were carried out. To test this hypothesis, apparently healthy subjects without detectable parasites by thick and thin blood smears within all age groups were recruited and both the total antibody response to recombinant MSP1_19 _and the fine specificity of anti-MSP1_19 _antibodies using a competitive ELISA with mAbs 12.8 and 12.10 were assessed. In a second objective, an effect of multiplicity of infection was examined. As carriage of multiple *P. falciparum *clones can be a useful indicator of the immune status of the host [28,29], the relationship between this parameter and the level of antibodies competing with the mAbs has also been investigated. Finally, the third objective of this work was to determine whether or not there was any association between the presence of specific invasion inhibitory antibodies, the anaemia status of the subject, and the carriage of multiple *P. falciparum *infections.

## Methods

### Study area

The cross-sectional study was conducted in Igbo-Ora, a rural area in south-western Nigeria; approximately 100 km from the state capital Ibadan. In Igbo-Ora malaria is hyperendemic with a prevalence of approximately 75% in children during the raining season from April to October and 55% to 60% during the dry season from November to March. The peak incidence was observed in November and August, with lower rates observed in July, September and October [30].

### Subjects and blood collection

Enrollment was carried out in September and October 2000. Asymptomatic carriers of a *P. falciparum *infection were defined as individuals with detectable blood stage parasites and an axilliary temperature <37.5°C, who had not received any anti-malarial drug in the preceding fifteen days and who did not present any malaria symptoms for at least seven days following blood sampling. Children homozygous for the sickle cell trait (HbSS) as well as children with HbSC were excluded from the study. The protocol was explained to the assembled village population and informed consent was obtained individually from each participant or his/her parents; the study was approved by the Ethical Committee of University College Hospital, Ibadan, Nigeria.

Blood was collected by venipuncture into heparinized and EDTA containing tubes. Thick and thin blood smears were stained with Giemsa for microscopic detection of malaria parasites, examining 200 fields. Malaria parasites were counted relative to leukocytes, and a leukocyte count of 8,000/μl of blood was assumed. Blood slides were examined using the quality control procedures described elsewhere [30]. Red blood cells were separated from the plasma by centrifugation and both were stored at -80°C prior to further analysis.

Haemoglobin type was determined by electrophoresis on cellulose acetate gel with alkaline buffer. Packed cell volume (PCV) was determined using a haematocrit centrifuge (Hawksley and Sons, Ltd., Lancing, United Kingdom). Anaemia was defined according to the age-dependent PCV levels published by the WHO (0-3 months: < 32%, 6 months-4 years: < 33%, 5-11 years: <34%, >12 years: < 37%; for adults, male: < 40% and female: <37%).

### Genotyping of *P. falciparum *msp1

DNA was extracted from blood cells using the QIAamp DNA blood mini kit (Qiagen) according to the manufacturer's procedure. Single and nested PCRs were performed to amplify the polymorphic sequence block 2 of *P. falciparum msp1 *as described previously [31]. Amplifications were performed in a final volume of 50 μl and cloned or monomorphic parasite lines were used as positive template controls. The first round PCR amplification was performed using two microlitres of DNA and the primers 5'-AAGCTTTAGAAGATGCAGTATTGAC-3' and 5'-ATTCATTAATTTCTTCATATCCATC-3', designed on the conserved regions in sequence blocks 1 and 3 on either side of block 2. The second round nested PCR amplification was carried out using 2 μl of the primary amplified products. Primers were used for detecting the K1 (5'-AAGAAATTACTACAAAAGGTG-3' and 5'-TGCATCAGCTGGAGGGCTTGCACCAGA-3'), RO33 (5'-AGGATTTGCAGCACCTGGAGATCT-3' and 5'-GAGCAAATACTCAAGTTGTTGCA-3') and MAD20 (5'-TGAATTATCTGAAGGATTTGTACGTCT-3' and 5'-GAACAAGTCGAACAGCTGTTA-3') *msp1 *block 2 sequence families [32]. The amplifications were performed in a Biometra Uno II thermal cycler (Biometra, Göttingen, Germany) and the products were loaded onto a 1.5% agarose gel (Peqlab Erlagen, Germany), electrophoresed and stained with ethidium bromide. The DNA was visualised under ultraviolet light and the fragment size polymorphism for each allelic family was determined. It was assumed that each PCR fragment represented at least one parasite genotype.

### MSP1_19 _recombinant protein

The recombinant *Pf*MSP1_19 _protein (rMSP1_19_) was prepared by standard techniques as a glutathione S-transferase (GST) fusion protein by expression from a pGEX-3X plasmid as described previously [33] and it represents the sequence of the Wellcome parasite line [34] (EMBL accession number X02919).

### Enzyme-linked immunosorbent assay (ELISA)

An indirect ELISA was performed to determine the prevalence and titre of IgG antibodies binding to rMSP1_19_, as previously described [16]. Ninety six well-polystyrene microtitre plates (Costar, Corning Inc. NY) were coated with 0.5 μg/ml rMSP1_19_. Following incubation with diluted plasma samples, bound antibodies were detected with horseradish peroxidase (HRP)-rabbit anti-human IgG conjugate (Dako, Denmark) and ABTS/hydrogen peroxidase substrate solution by measuring the absorbance at 650 nm. The plasma samples were diluted at a 1:25 ratio and then in two fold dilutions to 1:3200; the reciprocal end point titre (the highest dilution that gave an absorbance value greater than the background level measured with negative control samples) was log transformed, and data were expressed as geometric mean log reciprocal titres. The negative control samples were obtained from malaria-naïve residents of the United Kingdom.

### Competition ELISA assays

The ability of polyclonal antibodies in the human plasma samples to compete with mAbs 12.8 and 12.10 for binding to rMSP1_19 _was examined by competitive ELISA [22]. Initially each mAb was titrated against the rMSP1_19 _and then used in the competition assay at a concentration that gave an absorbance reading just below the maximal absorbance for that antibody (i.e. just below the top of the titration curve). Microplate wells were coated with 0.1 μg of rMSP1_19_, then plasma at a dilution of 1:50 or 1:250 was added to duplicate wells, and the plates were incubated overnight at 4°C. A predetermined amount of mAb was then added and incubation continued overnight; the amount of bound mAb was determined as described previously [22]. Plasma samples were divided into two groups: non-competitive samples, which were those that inhibited the mAb binding by less than 50% at a 1:250 dilution and competitive samples, which were those that inhibited the mAb binding by more than 50% at a 1:250 dilution.

### Statistical analysis

The multiplicity of infection (mean minimum number of different parasite genotypes per infected subject) was estimated by dividing the total number of *msp1 *PCR fragments representing different genotypes by the number of infected subjects. Infection was categorized as a nominal variable as follows: M (multiple, ≥ 2 parasite genotypes), S (single, 1 parasite genotype). The non-parametric Kruskall-Wallis and Mann-Whitney U tests were used to compare continuous variables between three (or more) and two groups, respectively. The chi square test was used to compare nominal data. The antibody response was divided into two groups: high competition/low mAb binding (≥50% inhibition of binding) and low competition/high mAb binding (<50% inhibition of binding). The effect of various parameters on a high or low competitive binding for both mAb 12.8 and 12.10 was evaluated by logistic regression. Statistical analysis of data was performed using the JMP 5 statistical software. Differences were considered statistically significant at a p value of less than 0.05.

## Results

### Characteristics of subjects

During the enrolment period, of 147 subjects recruited who had no detectable parasites by microscopy on Giemsa-stained thick and thin blood smears and who met the inclusion criteria, 143 had *P. falciparum *infections detectable by PCR with *msp1*-specific primers. Some characteristics of these subjects including sex, haemoglobin type, mean PCV and their use of bed and/or window nets are given in Table 1. Fifty nine percent of recruited individuals were found to be anaemic, particularly amongst younger subjects: 63% of less than 1 year-olds, 33% of 1 to 5 year-olds and 17% of 6 to 15 year-olds had PCVs below the WHO threshold, respectively. This decrease in anaemia with age was significant (p < 0.0001, r = 0.56).

**Table 1:** Characteristics of the subjects with *P. falciparum *sub-microscopic infections in Igbo-Ora village, Nigeria

Factor	Number of subjects	^**a**^**Sex (F/M)**	^**b**^**Nets (%)**	^**c**^**PCV**	^**d**^***P***
**Age (years)**					
<1	38	20/18	32 (84)	31.7 (6.8)	<0.0001
1 - 5	24	16/8	24 (100)	33.4 (2.7)	-
6 - 15	36	20/16	26 (72)	37.0 (3.7)	-
>15	49	37/12	38 (77)	39.5 (4.5)	-

**Haemoglobin phenotype**					
AA	135	85/50	111 (82)	34.7 (5.7)	ref
AS	11	7/4	8 (73)	38.2(4.7)	0.14
AC	1	1/0	1 (100)	30	nd

^e^**Minimum number of MSP1 genotypes**					
0 to 1*	74	48/26	62 (84)	34.6 (5.3)	0.86
2 to 3	68	41/27	54 (79)	35.0 (6.1)	-
4 to 5	5	4/1	4 (80)	35.8 (2.3)	-

**Total**	147	93/54	120 (82)	34.8 (5.6)	

^a ^Sex: F, female; M, male

^b ^Nets: indicates the use of bed and/or window nets; actual numbers and proportion of subjects (%).

^c ^PCV: mean packed cell volume (SD)

^d ^P value for differences in PCV; nd: not done

^e ^Number of MSP1 genotypes was determined by PCR, the presence of 1 or more indicates the presence of parasites.

*Four samples did not produce a PCR product with the MSP1 primers

### Prevalence of IgG antibodies reacting with rMSP1_19 _protein

Antibodies reacting with rMSP1_19 _were detected in 85% of the individuals with sub-microscopic infections. There was a significant increase in the proportion of responders to rMSP1_19 _protein (p < 0.0001) and in the mean log titre of anti-MSP1_19 _antibodies (Spearman correlation, p < 0.0001) with age, as shown in Table 2.

**Table 2:** Relationship between subjects' age, titre of anti-MSP1 antibodies, inhibition of binding of specific monoclonal antibodies and multiplicity of infection.

Age (years)	Total Number of Subjects	^a^Antibodies to rMSP1		^b^mAb12.10	mAb12.8	^c^Infection		
		
		Responders (%)	Mean Log titre	H (%)	H (%)	M	S	MOI
**<1**	38	23 (61)	2.21	7 (18)	8 (21)	14	23	1.5
**1 - 5**	24	20 (83)	2.35	2 (8)	7 (29)	12	11	1.8
**6 - 15**	36	34 (94)	2.69	33 (92)	13 (36)	27	8	2.2
**>15**	49	48 (98)	2.94	15 (31)	18 (37)	20	28	1.5

**Total**	147	125 (85)	2.60	57 (39)	46 (31)	73	70	1.7

^a^Antibodies to rMSP1: Responders is the number of subjects with antibodies against recombinant MSP1,(%) is the percentage of the total.

^b^mAb12.10 and mAb 12.8: H refers to the number of individuals with antibodies highly competitive with the mAb; (%) is the percentage of the total.

^c^Infection: M refers to individuals with two or more parasite clones; S refers to individuals with only one parasite type detected. MOI is the mean multiplicity of infection based on MSP1 block 2 alleles.

### Human antibodies that compete with mAbs 12.10 and 12.8 for binding to rMSP1_19_

There was marked heterogeneity in the ability of polyclonal antibodies to compete with the MSP1_19_-specific mAbs. The samples were divided into two groups: those containing highly competitive antibodies resulting in low binding of mAb to rMSP1_19 _(≥ 50% inhibition of binding), and those containing poorly competitive antibodies resulting in high binding of mAb to rMSP1_19 _(<50% inhibition of binding). As shown in Table 2, 39% (57 of 147) of samples contained antibodies that were highly competitive to mAb 12.10 binding to rMSP1_19_. The 1- to 5 year-old age group contained the lowest prevalence of antibodies competitive with mAb 12.10 and although the inhibition of mAb 12.10 binding varied between age groups the difference was not significant. The inhibition of mAb 12.10 binding correlated strongly with the prevalence of total anti-MSP1_19 _antibodies (Spearman correlation test, p < 0.0001) and with the mean titre of anti-MSP1_19 _antibodies (Spearman correlation test, p < 0.001) (Table 3). Forty-six of the 147 samples (31%) contained antibodies that competed with mAb 12.8, and there was a correlation between the presence of such antibodies and age (p < 0.001). There was also a strong correlation of competition with mAb 12.8 and the prevalence and mean titre of polyclonal anti-MSP1_19 _antibodies (p < 0.0001) (Tables 2 and 3). Overall, plasma samples containing antibodies highly competitive with both mAbs were also those with the highest titres of total anti-MSP1_19 _antibodies (Table 3).

**Table 3:** The multiplicity of *P. falciparum *infection and mean titer of anti-MSP1 antibodies, in subjects with antibodies that are either highly competitive (H) or poorly competitive (L) with the specific monoclonal antibodies.

^a^Monoclonal antibody	Number of subjects		^c^Infection			^d^Antibodies to rMSP1			
	
	Total n = 147	^b^Infected	M	S	MOI	Prevalence	*P*	Mean log titre	*P*
**12.8**									
Poorly competitive (L)	100	99	50	49	1.73	78%	0.0006	2.3	0.0001
Highly competitive (H)	46	43	22	21	1.65	100%		3.2	

**12.10**									
Poorly competitive (L)	90	87	38*	49	0.86	77%	0.002	2.4	0.0001
Highly competitive (H)	57	56	35	21	0.98	96%		2.9	

**12.8 and 12.10**									
Poorly competitive (L)	75	74	33 **	41	1.62	73%	0.0007	2.3	0.0001
Highly competitive (H)	32	31	18	13	1.77	100%		3.2	

^a^Monoclonal antibody. mAb12.10 and mAb 12.8: L and H refer to the number of individuals with antibodies that are either poorly competitive or highly competitive with the monoclonal antibodies, respectively.

^b^Infected: number of subjects positive by PCR.

^c^Infection: M refers to individuals with two or more parasite clones, S refers to individuals with only one parasite type detected. MOI is the mean multiplicity of infection based on MSP1 block 2 alleles.

^d^Antibodies to rMSP1: Prevalence is the number of subjects with antibodies against recombinant MSP1 as a percentage of the total.

### Genetic diversity in msp1 sequence block 2 and multiplicity of infection

**Figure 1:**
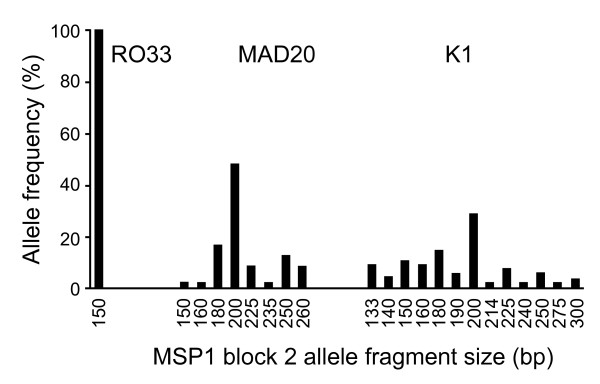
**Distribution and prevalence of MSP1 alleles within sequence block 2 that were identified in the cross sectional survey**. The different alleles fall into 3 different sequence families (RO33, MAD20, and K1) within which there are size polymorphisms identified by the size of the PCR amplified fragment (shown in base pairs, bp)

It was of interest to determine whether or not multiplicity of infection (MOI), and the numbers of different MSP1 types affected either the total amount of MSP1_19_-specific antibody or of antibody that competed with the mAbs, and therefore the number of *msp1 *sequence block 2 types in the samples was used as a marker for MOI and MSP1 polymorphism in each individual. Genotyping of the polymorphic sequence block 2 revealed that RO33 was the predominant allele with 57% of the total, with the K1 and MAD20 allelic families representing 26% and 17%, respectively. Based on size polymorphism, only one RO33 allele was detected whereas the MAD20 and K1 families contained 8 and 13 distinct alleles, respectively (Figure 1). There was no preferential distribution of allelic families between age-groups. The mean minimum MOI based on the number of *msp1 *genotypes detected for individuals in each age-group is shown in Table 2. The highest MOI was found in the 6 to 15 years age group. Seventy-three of 147 (51%) isolates were found to have more than one *P. falciparum *genotype. There was no significant influence of age on the proportion of individuals with multiple versus single infections (denoted by M and S, respectively in Table 2) or on MOI. Overall, no correlation between MOI and the mean log titre of anti-MSP1_19 _antibodies was detected. Similarly the MOI was not correlated with inhibition of mAb 12.10 binding. However, comparing the two groups M (multiple infection) and S (single infection), there was a higher prevalence of antibodies that competed with mAb 12.10 in samples from group M (Table 3, p = 0.04).

### Relationship between MOI, anaemia, and the inhibition of mAb binding

**Figure 2:**
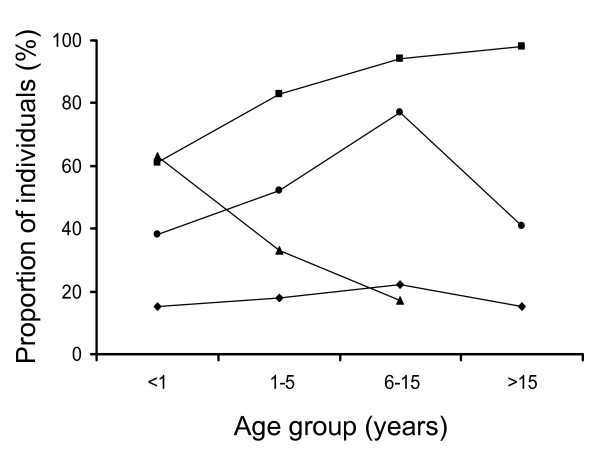
**Prevalence of individuals with antibodies reacting with recombinant MSP1, with anaemia, with infection with two or more parasite clones, and with multiplicity of infection (MOI), for the different age-groups**.

The proportion of individuals with anaemia decreased with age (Figure 2). Using logistic regression, the association of anaemia and the carriage of multiple *P. falciparum *genotypes on the total MSP1_19_-specific antibody and the competitive activity with mAbs 12.10 and 12.8 were examined. Anaemia was significantly associated with total anti-MSP1_19 _antibody (p = 0.0016) with a low prevalence of antibodies that competed with mAb 12.10; whereas harbouring more than 1 parasite genotype (group M) was associated (p = 0.04) with a greater prevalence of antibodies that competed with mAb 12.10. This was not observed with MAb 12.8: the presence of antibody competitive with mAb 12.8 was influenced neither by the presence of multiple *P. falciparum *genotypes nor by anaemia.

## Discussion and Conclusions

The first candidate malaria vaccines based on MSP1 are now being evaluated in clinical studies [35]. However, in view of the disappointing results in the first MSP1 clinical trial in Kenya [36], there is an urgent need to define useful correlates of protective immunity and surrogate assays to evaluate malaria vaccines based on MSP1_19 _and other parts of the molecule.

The two mAbs 12.10 and 12.8, which inhibit the invasion of red blood cells and have overlapping epitopes [26], have been found to be useful in field studies to discriminate the potential contribution of each specificity to protection against clinical malaria or high-grade parasitaemia [21,23]. Asymptomatic carriage of parasites by young children as well as adults [37] reflects their ability to control the malaria infection through the presence of effective innate or acquired immune mechanisms. Therefore it was proposed that in the samples from such individuals, with a parasitaemia undetectable by microscopy, that there would be antibodies which would be highly effective at inhibiting parasite invasion of red blood cells, as reflected by higher competition with the mAbs 12.8 and 12.10 for binding to MSP1_19_. In addition, it was of interest to evaluate whether or not there is an association between the level of antibodies that inhibit red blood cell invasion (assessed indirectly by the competition with the mAbs) and the carriage of sub-microscopic infections comprised of multiple parasite clones, and representing an antigenically diverse parasite population.

In our Nigerian cohort, 85% of plasma samples contained anti-MSP1_19 _antibodies. Both the prevalence and the mean titre of anti-MSP1_19 _antibodies were significantly correlated with the age of the donor. This finding differs from that in a previous report from the same area [16], which noted a decline in the MSP1_19_-specific antibody titre with age in samples collected in the rainy season, whereas in samples collected in the dry season higher antibody titres were observed for children of six years of age or older than for younger children. In studies carried out in the Gambia and Uganda, the prevalence and mean anti-MSP1_19 _antibody levels did not increase with age [21,23]. This discrepancy between the studies may be explained by differences in the intensity of malaria transmission, in the time of sample collection (seasonal variation) or in study design. Indeed, in the Gambian and Ugandan studies, individuals were followed up for some weeks and the objective was to search for association between outcome of the infection and reinfection and high parasite load. In our cross-sectional survey, parasitaemia was a controlled parameter.

In the competition ELISA, 39% and 31% of samples contained antibodies that strongly inhibited the binding of mAb 12.10 and mAb 12.8, respectively. The inhibition of mAb 12.8 binding correlated positively with the titre of total anti-MSP1_19 _antibodies as also found in Uganda, but not in The Gambia [23]; no influence of the age of the donor was found in these studies. Whilst competition with mAb 12.10 also correlated positively with total anti-MSP1_19 _antibody titres in the Nigerian samples, there was no significant change in the prevalence of these competing antibodies with age as observed in the other African sites [21,23].

The mAbs 12.8 and 12.10 are antibodies that inhibit the secondary processing of MSP1 as well as the invasion of red blood cells. Previously this functional antibody activity was detected in samples similar to those we report on here [16] and this activity did not correlate with the titre of total anti-MSP1_19 _antibody. Although a competition ELISA assay with mAbs 12.8 and 12.10 might be expected to provide a surrogate read-out for this function the situation is complicated by the potential presence of so-called blocking antibodies that compete with 12.8 and 12.10, but do not themselves inhibit MSP1 processing or erythrocyte invasion [24].

In malaria endemic regions, anaemia has multiple causes e.g. polyparasitism, poor nutrition or immune depression. It can also result from both acute and chronic malaria [38]. A high MOI is considered to reflect acquired immunity or premunition [39] and to reduce the risk of clinical malaria and morbidity [28,29], in part by selection against virulent parasites. However, May *et al *[40] showed that a high multiplicity of *P. falciparum *infection can be associated with an increased risk of anaemia. Here, in infants and older children with sub-microscopic infection, no association was found between MOI and anaemia status. However, the multiplicity of infection may have been underestimated [29] because only one blood sample was collected per individual and fluctuations in sequestered parasite populations and the sensitivity of the PCR method [41] may limit detection at low parasitaemia. In addition, the use of a single genetic marker can only provide a minimum estimate of diversity in the parasite population.

Immune responses (e.g. production of various cytokines) that vary with different parasite strains may contribute to the unequal impact of distinct parasite genotypes on haemoglobin levels.

An evaluation of the association of non severe malarial anaemia and *P. falciparum *polyclonal infections on the prevalence of antibodies which compete with mAb 12.10 showed that anaemia and carriage of multiple infections had a negative and a positive association, respectively. This association was not found for the prevalence of antibodies competing with mAb 12.8 binding. Interestingly, the possession of anti-MSP1_19 _antibodies that compete with mAb 12.10 and not 12.8 has been associated with resistance to high-density parasitaemia [21,23]. These observations support not only the proposed importance of antibody fine specificity in playing a major role in protection against parasite load but also suggest a differential contribution of the overlapping specificities identified so far.

## List of abbreviations

GPI: glycosylphosphatidyl inositol; GST: glutathione S-transferase; HRP: horse radish peroxidise; MSP: Merozoite Surface Protein; mAb: monoclonal antibody; MOI: multiplicity of infection

## Authors' contributions

J N-L, RIN, FN, MN, CIA, AA, WDMM, and AAH made substantial contributions to the conception and the design of the study, or acquisition, analysis and interpretation of data; HOA and ABO were involved in drafting the manuscript or revising it critically for content; and RIN, AAH and FN gave final approval of the version to be published; all authors read and approved the final manuscript.
